# Current Management of Acute Severe Ulcerative Colitis: New Insights on the Surgical Approaches

**DOI:** 10.3390/jpm14060580

**Published:** 2024-05-28

**Authors:** Sara Lauricella, Francesco Brucchi, Federica Cavalcoli, Emanuele Rausa, Diletta Cassini, Michelangelo Miccini, Marco Vitellaro, Roberto Cirocchi, Gianluca Costa

**Affiliations:** 1 Colorectal Surgery Division, Department of Surgery, Fondazione IRCCS Istituto Nazionale dei Tumori, 20133 Milan, Italy; 2 University of Milan, 20122 Milan, Italy; 3 Gastroenterology and Endoscopy Unit, Fondazione IRCCS Istituto Nazionale dei Tumori, 20133 Milan, Italy; 4 General and Emergency Surgery, Sesto San Giovanni Hospital, 20099 Milan, Italy; 5 Department of General Surgery, Sapienza Medical School, 00185 Rome, Italy; 6 Digestive and Emergency Surgery Unit, S. Maria Hospital Trust, 05100 Terni, Italy; 7 Department of Life Science, Health, and Health Professions, Link Campus University, 00165 Rome, Italy

**Keywords:** ulcerative colitis, acute severe ulcerative colitis, medical management, salvage therapy, colorectal surgery, IPAA, transanal approach, functional outcomes

## Abstract

Acute severe ulcerative colitis (ASUC) is a life-threatening medical emergency with considerable morbidity. Despite recent advances in medical IBD therapy, colectomy rates for ASUC remain high. A scoping review of published articles on ASUC was performed. We collected data, such as general information of the disease, diagnosis and initial assessment, and available medical and surgical treatments focusing on technical aspects of surgical approaches. The most relevant articles were considered in this scoping review. The management of ASUC is challenging; currently, personalized treatment for it is unavailable. Sequential medical therapy should be administrated, preferably in high-volume IBD centers with close patient monitoring and indication for surgery in those cases with persistent symptoms despite medical treatment, complications, and clinical worsening. A total colectomy with end ileostomy is typically performed in the acute setting. Managing rectal stump is challenging, and all individual and technical aspects should be considered. Conversely, when performing elective colectomy for ASUC, a staged surgical procedure is usually preferred, thus optimizing the patients’ status preoperatively and minimizing postoperative complications. The minimally invasive approach should be selected whenever technically feasible. Robotic versus laparoscopic ileal pouch–anal anastomosis (IPAA) has shown similar outcomes in terms of safety and postoperative morbidity. The transanal approach to ileal pouch–anal anastomosis (Ta-IPAA) is a recent technique for creating an ileal pouch–anal anastomosis via a transanal route. Early experiences suggest comparable short- and medium-term functional results of the transanal technique to those of traditional approaches. However, there is a need for additional comparative outcomes data and a better understanding of the ideal training and implementation pathways for this procedure. This manuscript predominantly explores the surgical treatment of ASUC. Additionally, it provides an overview of currently available medical treatment options that the surgeon should reasonably consider in a multidisciplinary setting.

## 1. Introduction

Ulcerative colitis (UC) is a chronic inflammatory bowel disease characterized by the episodic occurrence of hematochezia and diarrhea, which can lead to considerable disability and a diminished quality of life (QoL) [1].

Approximately 12–25% of all ulcerative colitis patients will experience at least one severe acute exacerbation during their lifetime, often occurring at disease onset and necessitating hospitalization [2,3]. An episode of acute severe UC (ASUC) can be a life-threatening medical emergency with an overall mortality of 1% [4]. In severe flares, ASUC patients may experience weight loss, fevers, and malaise [1].

Little is known about ASUC pathogenesis: the condition may result from a dysregulated systemic immune response to commensal pathogens in genetically predisposed individuals, highlighting the intricate interplay between genetic susceptibility and immune dysfunction [5,6]. Microorganisms have been postulated as potential instigators of ASUC due to observed parallels with infectious colitis, notably the manifestation of systemic inflammation. Furthermore, cytomegalovirus or Clostridioides difficile has been detected in 10–30% of individuals diagnosed with ASUC [7,8].

For several decades, intravenous corticosteroids have consistently represented the primary and standard initial medical intervention. However, approximately 40% of patients demonstrate an inadequate response to this treatment, necessitating a secondary line of therapeutic intervention [9,10].

In the past, failure to achieve clinical remission with intravenous corticosteroids typically resulted in colectomy. However, the introduction of medical rescue or salvage therapies for steroid-refractory cases has offered an alternative option to surgery [11].

Infliximab and ciclosporin have been demonstrated as effective second-line therapies, as evidenced by a randomized controlled trial (RCT) by Laharie et al. [12].

In cases where cyclosporine or infliximab fail to induce remission, salvage therapy with either cyclosporine or infliximab may be considered to avoid colectomy. Recent advancements in UC management include the introduction of new drugs like vedolizumab, ustekinumab, and tofacitinib, which are also being explored for ASUC. However, the efficacy and safety of salvage therapies remain largely unknown [13].

Currently, personalized treatment for ASUC is unavailable. Treatment involves sequential medical therapy with close monitoring to determine the need for colectomy if medical treatment fails or if a complication occurs. 

This paper will predominantly focus on the surgical aspects of managing ASUC. It will initially cover the updated general principles applicable in the biological era and then shift its emphasis towards exploring surgical interventions for ASUC, examining the evidence supporting various surgical strategies in its management.

## 2. Methods

This is a scoping review of published data on ASUC. We used PubMed and Cochrane databases and we searched for the following terms: “ulcerative colitis”; “acute severe ulcerative colitis”; “medical management”; “salvage therapy”; “colorectal surgery”; “IPAA”; “transanal approach”; “functional outcomes”. Electronic databases were consulted until April 2024 for relevant publications. Inclusion criteria were original scientific manuscripts, review articles, meta-analyses, case reports, English-language studies, and articles focused on medical treatment options and surgical management of ASUC. No limitation was applied to study type, sample size and patient’s age. Exclusion criteria were articles unrelated to the review topic. SL and FB double-screened titles and abstracts and checked full texts against eligibility criteria. Disagreements were resolved by discussion or, if unresolved, through arbitration with a third reviewer (RC). We collected data, such as general information of the disease, diagnosis and initial assessment, available medical and surgical treatments focusing on technical aspects of surgical approaches. The most relevant articles were considered in this scoping review.

## 3. Diagnosis and Initial Assessment

The diagnosis of ASUC is based on the Truelove and Witts criteria, which evaluate the number of bloody stools per day and the presence of a systemic inflammatory response. This score is linked to the risk of colectomy at days 3 and 5 [6].

Investigations at admission should include complete blood test with kidney function, albumin, hemoglobin, CRP, and pre-therapeutic evaluation with viral serologies and tuberculosis screening, stool culture and tests for C. difficile toxins and flexible sigmoidoscopy with biopsies and CMV immunohistochemistry testing [14].

In addition, early and repeated imaging (CT scan and/or ultrasound) should be performed to look for complications such as abscesses, perforation, and toxic megacolon. Indeed, patients presenting with toxic megacolon, described as a colonic dilatation larger than 6 cm, are at high risk of perforation and should be referred to a surgeon for emergent colectomy.

The risk of thromboembolism is high in patients with a flare of UC and requires appropriate prophylaxis with low molecular weight heparin. The use of antibiotics is not routinely recommended in ASUC in the absence of complications or suspected sovra-infection [15].

Triggering and risk factors should be investigated. Non-steroidal anti-inflammatory drugs (NSAIDs) represent potential exacerbating factors, and their intake should be carefully evaluated. Enteric infections have been reported as a risk factor. In individuals with inflammatory bowel disease (IBD), Clostridioides difficile-related colitis has been linked to increased morbidity and mortality. Cytomegalovirus (CMV) is associated with higher colectomy rates in acute colitis [16,17].

In managing ASUC, nutrition care is integral to the therapeutic approach. Malnutrition affects up to 62% of patients with UC and is associated with worse prognosis, higher complication rates, decreased QoL, and increased mortality risk [18].

Therefore, correction of altered body composition, iron deficiency, and nutrition imbalances are advised preoperatively. During an ASUC episode, enteral nutrition (EN) may be used in combination with intravenous steroids as part of the management strategy [19].

Exclusive enteral nutrition (EEN) has been shown to potentially augment corticosteroid responsiveness in patients with ASUC, leading to a lower rate of corticosteroid failure, a shorter hospital stay, and improvements in inflammatory markers such as serum C-reactive protein and fecal calprotectin levels [20]. Additionally, EEN has been associated with beneficial alterations in the gut microbiome, which may contribute to its therapeutic effects [21]. However, the most recent guidelines must address EN’s role in managing ASUC [22]. The decision to use EN should be individualized based on the patient’s clinical status and nutritional needs.

A staged procedure is often preferred for those patients who are nonresponsive to medical treatment and who can have their surgery planned electively. This entails enhancing patient status and minimizing postoperative complications [23]. Corticosteroids should be weaned before surgery as patients taking high prednisone doses for four weeks or longer are at increased risk of anastomotic leak and pelvic sepsis, which are leading causes of pouch failure [24]; otherwise, it is advisable to postpone pouch construction [25].

Preoperative thiopurine or cyclosporine does not increase the risk of postoperative complications [24]. Conversely, patients on biologics might be at increased risk of developing early and late pouch-specific complications, but study quality is low [26].

## 4. Medical Management

### 4.1. First-Line Therapy

Since the 1950s, intravenous steroid therapy has been the cornerstone of ASUC management [6].

Current guidelines recommend IV corticosteroids (0.8–1 mg/kg) for 5–7 days as the first-line treatment for ASUC [25]. Truelove and Witts’ historic trial showed a 41% remission rate and 7% mortality with IV steroids, compared to 16% remission and 24% mortality in the placebo group [6]. A 2007 meta-analysis revealed a 67% short-term (within one week) response to steroids [10].

Recent findings suggest that tofacitinib could potentially replace intravenous steroids as first-line therapy for ASUC, showing similar effectiveness and safety to oral steroids in inducing remission in a randomized pilot trial with patients having moderately active ulcerative colitis [27].

Anticipating steroid failure either before initiating or shortly after beginning steroid treatment could enable clinicians to promptly transition to second-line therapies, thereby optimizing the management of ASUC. Various parameters and scores have been devised to predict the clinical response to first-line treatment.

The Oxford criteria, the Ho index, and the Swedish fulminant colitis index define intravenous steroid failure as early as day 3 of therapy. In contrast, the Seo index is based on measures taken each week [23,28,29,30]. The Oxford criteria, based on a 1996 study of 49 ASUC patients, aimed to predict colectomy risk during the third to fifth day of steroid treatment. This method considers critical parameters such as the frequency of bowel movements and C-reactive protein (CRP) levels. Specifically, the criteria indicate that the persistence of more than eight bowel movements or 3–8 stools and a CRP level exceeding 45 mg/L at day three is associated with a substantial 85% risk of undergoing colectomy [23]. However, a 2017 study reported a colectomy rate of 36%, deviating from the historically high 85%. This shift highlights the changing standard-of-care practices, making the Oxford criteria less relevant in contemporary ASUC management.

While these scores help predict specific ASUC outcomes, they should not replace a comprehensive assessment for guiding decisions on surgery or second-line therapies.

### 4.2. Second-Line Therapy

In steroid-refractory patients, second-line medical therapy, including calcineurin inhibitors (CNIs) and infliximab, should be considered [24]. Close monitoring is crucial, and colectomy should not be delayed, if necessary, for the patient’s well-being. Swift decision-making is essential in such cases.

#### 4.2.1. Infliximab

The introduction of infliximab for ASUC treatment dates back 20 years and has proven a significant advantage in this setting. In 2005, a randomized, placebo-controlled study demonstrated the efficacy of infliximab for ASUC in avoiding in-hospital colectomy (71% vs. 33% in the placebo group, *p* = 0.017) without relevant differences in adverse vents rate between the groups [31]. Subsequently, additional studies provided further support for the use of infliximab as the drug of choice for salvage therapy [32].

The infliximab administration follows the standard induction and maintenance protocol with 5 mg/kg infused at weeks 0, 2, and 6 and then every eight weeks. Although some studies [33] support the efficacy of infliximab dose escalation in patients with ASUC, the PANCCO Clinical Practice Guidelines recommend against the routine use of an intensified regimen [34].

A systematic review and meta-analysis found that multiple 5-mg/kg infliximab doses are superior to single-dose salvage. Still, dose intensification with either high-dose or accelerated strategies did not show a significant difference compared to 5-mg/kg standard induction at three months [35].

Additionally, the Korean Clinical Practice Guidelines report mixed results from retrospective observational studies on the benefit of accelerated over standard infliximab dosing in ASUC patients, with some studies showing no difference in colectomy rates and others suggesting a benefit in selected patients with more severe disease [36].

In summary, while some evidence suggests potential benefits of dose-escalated infliximab in ASUC, the current guidelines and systematic reviews do not strongly support routine intensified dosing regimens. The decision to use such an approach should be individualized based on the patient’s disease severity and response to standard induction therapy.

#### 4.2.2. Cyclosporin

Before the era of biologics, ciclosporin served primarily as a bridge therapy to azathioprine in ASUC [37]. In terms of short-term usage, a randomized controlled trial (RCT) involving 73 patients, comparing different cyclosporine doses, found similar response rates at day 8 for both a 4 mg/kg/day dose (84.2%) and a 2 mg/kg/day regimen (85.7%) [37]. However, the 4 mg/kg group exhibited a trend toward higher blood pressure and nephrotoxicity. Consequently, current guidelines recommend a more conservative 2 mg/kg/day regimen with drug-level monitoring [38]. This approach seeks to balance therapeutic efficacy with mitigating potential adverse effects. 

Comparison trials [12] found no significant difference in immediate (day 7) and short-term outcomes (day 98) between infliximab and ciclosporin in treating steroid-refractory patients, as shown in the CySIF randomized controlled trial involving 115 participants. In a long-term follow-up of the CySIF study (median 5.4 years [IQR 4.7–6.2]), there was no significant difference between groups in colectomy-free survival at one year (71% (95% CI 59–83) with ciclosporin vs 69% (57–81) with infliximab) and five years (61% (49–74) vs. 65% (52–78)) [39].

Ciclosporin appears more suitable for patients without associated comorbidities, such as renal impairment. In thiopurine-naive patients, ciclosporin is a bridging agent for thiopurine maintenance therapy [19]. In patients with extraintestinal manifestations like ankylosing spondylitis or psoriasis, infliximab is preferred over ciclosporin due to its effectiveness in managing extraintestinal inflammation in individuals with inflammatory bowel disease (IBD) [40].

#### 4.2.3. New Molecules

Hospital admissions for ASUC patients unresponsive to infliximab and other biologics are increasing, prompting the need for alternative non-anti-TNF biologics and small-molecule rescue therapies.

Vedolizumab (humanized IgG1 monoclonal antibody targeting integrin α4 β7) has been approved by regulatory agencies like the European Medicines Agency and the US Food and Drug Administration for treating moderate-to-severe ulcerative colitis. The onset of action of vedolizumab may be slow, with response and remission rates increasing over the first ten weeks of treatment. However, this speed of action is inadequate for treating ASUC [41].

Limited data exist on ustekinumab’s efficacy in ASUC, with only three retrospective studies involving 13 patients [42]. All were steroid-refractory and had previously failed anti-TNF and vedolizumab treatments. Sequential therapy with a calcineurin inhibitor followed by ustekinumab avoided colectomy in all cases. Serious adverse events and mortality related to ustekinumab were both 0%, suggesting potential efficacy and safety in ASUC. However, due to the scarcity of data, conclusions are difficult to draw [43].

Tofacitinib, an oral JAK-STAT pathway inhibitor, is approved for moderate-to-severe ulcerative colitis. It quickly absorbs and shows efficacy over placebo within three days. Its rapid clearance minimizes risks during emergency colectomy or rescue therapy, making it an attractive option in ASUC [44,45].

The effectiveness of newer, more selective JAK inhibitors in ASUC remains uncertain. Limited data are available for filgotinib, but a case series involving six ASUC patients who did not respond to infliximab showed favorable outcomes with upadacitinib, with only one patient requiring colectomy after 15 weeks [46].

### 4.3. Third-Line Therapy (Salvage Therapy)

In the context of sequential therapy for ASUC, the pivotal consideration revolves around weighing the benefits against the risks between surgery and sequential therapy. Indeed, postponing urgent surgery to the elective setting could also be a favorable outcome of such sequential treatment. If second-line therapy proves ineffective, referral to expert inflammatory bowel disease (IBD) centers is advisable. 

A small retrospective study examined two sequences: nine patients receiving cyclosporine after infliximab and ten undergoing the opposite sequence. Remission rates were 40% in the cyclosporin-to-infliximab group and 33% in the alternate group, showing no statistically significant difference [47].

In a separate retrospective study of 86 patients treated sequentially with infliximab and ciclosporin, 57% did not respond to the second-line salvage treatment and opted for colectomy. Adverse events included infections and one death in the cyclosporin-to-infliximab group [48].

Initially, guidelines were cautious about recommending a second line of salvage therapy, fearing potential delays in colectomy [49,50]. A 2016 systematic review of 314 patients across ten studies reported a 62.4% overall short-term response rate after sequential treatment, with similar colectomy rates between different salvage therapies at three months and one year. Adverse events occurred in 23% of patients, including severe infections and a 1% mortality rate [51].

As a result, third-line medical therapy in ASUC has been deemed a limited option for highly selected patients and should be restricted to expert IBDs. Nevertheless, colectomy remains the standard of care for third-line management, as the European Crohn’s and Colitis Organization (ECCO) guidelines recommended [25]. This underscores the nuanced approach required for sequential therapies in managing ASUC.

## 5. Surgical Management

Despite recent advances in medical IBD therapy and the reduction in overall mortality over time, colectomy rates for ASUC remain high [52]. Approximately 15% of UC patients develop an acute attack of severe colitis, and up to 40% of these patients fail to respond to conservative treatments and require a colectomy [10,53,54,55].

Colectomy is highly recommended in case of acute severe ulcerative colitis whose condition worsens or is nonresponsive to rescue therapy after 5–7 days, or if complications occur (e.g., toxic megacolon, perforation, dysplasia or cancer, refractory bleeding, and persistent malaise) [24,56,57]. However, emergency procedures are associated with high morbidity, increased hospital costs, poor postoperative outcomes, and have a negative impact on patients’ quality of life [58,59].

A multidisciplinary team involving the colorectal surgeon and the gastroenterologist is crucial to identify the best time to stop medical treatment when necessary and proceed with surgery.

### 5.1. Emergency

Emergency surgery entails total colectomy and end ileostomy as a life-saving procedure. Managing rectal stump is challenging, and all individual and technical aspects should be considered. Although the risk is low, the retained rectum may be a site of potential cancer and cause refractory bleeding. In an emergency, the rectal stump may be managed by implanting it in the subcutaneous tissues, creating a mucosal fistula, or decompressing it transanally via a rectal tube. This approach minimizes the risk of rectal stump dehiscence and effectively manages the patient’s acute condition. Surgical options include progression to restorative proctocolectomy if suitable, or completion proctectomy. In elderly patients with significant rectal disease who are not fit for pouch construction or ileorectal anastomosis, the mucosectomy technique may be a valid option. 

### 5.2. Elective Surgical Options

In the case of elective procedures, surgical options include panproctocolectomy and end ileostomy, total colectomy and ileorectal anastomosis, or panproctocolectomy and ileal pouch–anal anastomosis. The choice is based on clinical criteria, patient preference, and anal sphincter function. 

However, when performing colectomy for ASUC, the traditional three-step approach consists of sub-total colectomy with ileostomy first with the rectum left in-situ, followed by a reconstructive surgery 3–6 months later with ileal pouch–anal construction with a defunctioning loop ileostomy and a final operation with ileostomy closure. The modified two-stage ileal pouch–anal anastomosis (IPAA) approach, which consists of a subtotal colectomy followed by completion proctectomy and IPAA without a diverting ileostomy, thus avoiding a third operation, has been increasingly utilized and compared to the traditional two-stage and three-stage approaches [24].

In both cases, the second step is usually performed a few weeks after colectomy, as patients are often malnourished, debilitated, and exhausted. The modified two-stage IPAA is not associated with increased rates of anastomotic leak, pelvic sepsis, or pouch failure when compared with the conventional two-stage IPAA [60]. A retrospective study conducted by Swenson et al. found that the modified two-stage approach had comparable functional outcomes to the three-stage approach in terms of bowel movements and fecal incontinence, but lower hospital costs and shorter hospital stays [61]. Luo et al. conducted a systematic review and meta-analysis indicating that the modified two-stage approach may be safe for adult patients, potentially reducing costs and length of stay, especially in those with lower preoperative biologic use rates [62].

The shift towards a modified two-stage procedure may become a standard of care, replacing one-, two-, and three-stage IPAA [63,64,65].

The modified two-stage IPAA may offer a safe and cost-effective alternative to the traditional two-stage and three-stage approaches for adult patients, particularly those with lower exposure to biologics. However, the three-stage approach may still be preferable in pediatric patients and those with higher preoperative biologic use [62]. It is essential to individualize treatment, considering disease severity, preoperative exposure to immunomodulators, comorbidities, anemia, nutritional status, and intraoperative factors.

Total proctocolectomy with permanent end ileostomy is also a reasonable option for some patients who present contraindications to IPAA. However, studies have shown that patients undergoing total proctocolectomy with end-ileostomy are at increased risk of developing ileostomy prolapse, parastomal hernia, and costs related to ileostomy supply [66,67,68,69,70].

## 6. Technical Aspects of Surgical Approaches

### 6.1. Stapled vs. Hand-Sewn Anastomosis

Stapled IPAA is associated with a lower rate of anastomotic stricture, small bowel obstruction, and ileal pouch failure compared to hand-sewn IPAA [71]. Additionally, patients undergoing stapled IPAA have reported better outcomes in terms of seepage per day and by night, pad use, night incontinence, resting pressure, and squeeze pressure in manometry evaluation [71].

Conversely, hand-sewn IPAA has been associated with a more significant proportion of patients describing incontinence, seepage, pad usage, and dietary, social, and work restrictions [72].

Furthermore, mucosectomy with hand-sewn anastomoses has been linked to higher median Wexner scores, indicating poorer fecal continence outcomes [73].

However, it is essential to note that some studies have found no significant difference in the long-term quality of life (QoL) between hand-sewn and stapled IPAA, suggesting that both techniques result in similar QoL in the late postoperative period [74].

In summary, stapled IPAA tends to have better functional outcomes and a lower incidence of specific complications than hand-sewn IPAA. However, long-term QoL may be similar between the two techniques.

### 6.2. Laparoscopic or Open Surgery?

Minimally invasive surgery is the preferred approach in most referral centers whenever feasible. In the context of ASUC, the decision between laparoscopic and open surgery must be individualized, considering the patient’s condition, the surgeon’s expertise, and institutional resources. Despite longer operative times, laparoscopic surgery is associated with shorter postoperative recovery and lower overall complication rates compared to open surgery [75,76,77].

The most recent guidelines recommend a minimally invasive surgical approach when expertise is available and appropriate, based on high-quality evidence from randomized controlled trials and large database studies [78].

In summary, while laparoscopic surgery for ulcerative colitis may offer certain postoperative recovery advantages, the surgical approach should be tailored to the individual patient, considering the available evidence and surgical expertise.

### 6.3. Robotic versus Laparoscopic Ileal Pouch–Anal Anastomosis (IPAA)

In comparing short-term postoperative outcomes following robotic versus laparoscopic ileal pouch–anal anastomosis (IPAA), the current medical literature suggests that both approaches yield similar outcomes in terms of safety and postoperative morbidity. Panteleimonitis et al. found no statistically significant differences in short-term outcomes between the two methods, with a trend towards a shorter stay for robotic IPAA. However, this was not statistically significant [79]. Similarly, Lightner et al. reported equivalent 30-day postoperative outcomes for both laparoscopic and robotic IPAA, with no differences in rates of superficial surgical site infection, peri-pouch abscess, anastomotic leak, pelvic abscess, readmission, or reoperation [80]. Opoku et al. also found that the operative approach (open, laparoscopic, or robotic) was not associated with better short-term outcomes, including length of stay, overall morbidity, anastomotic leak, reoperation, the incidence of ileus, and 30-day readmissions, in the context of ileal pouch–anal anastomosis [81].

Gebhardt et al. indicated that robotic-assisted proctectomy with IPAA can be performed with comparable short-term clinical outcomes to laparoscopy. Still, it is associated with a longer operative time and higher surgery costs [82].

These findings suggest that while robotic IPAA is feasible and safe, it may not confer significant short-term advantages over the laparoscopic approach regarding clinical outcomes. However, the potential for reduced length of hospital stay with robotic surgery may warrant further investigation with larger-scale studies to confirm these findings [79,80,82].

### 6.4. The Transanal Approach to Ileal Pouch–Anal Anastomosis (Ta-IPAA)

The transanal approach to ileal pouch–anal anastomosis (Ta-IPAA), also known as the TaPouch procedure, is a recent surgical technique for creating an ileal pouch–anal anastomosis via a transanal route. This approach is an adaptation of the transanal total mesorectal excision (TaTME) technique used for rectal cancer and has been applied to restorative proctocolectomy for conditions such as ulcerative colitis [83,84,85,86,87,88,89,90,91].

The Ta-IPAA procedure involves a transanal dissection of the rectum, allowing for a precise identification of the rectotomy site, which helps to avoid a long rectal stump and facilitates a single stapled anastomosis [85]. This technique has been reported to provide good visualization and access during pelvic dissection, potentially translating to lower conversion rates and similar or lower odds of postoperative morbidity compared to other minimally invasive techniques [86].

Functional outcomes after Ta-IPAA have been reported as satisfactory in the short term, with quality-of-life measures such as the Cleveland Global Quality of Life and Oresland Score being used to assess these outcomes [84,89]. However, the impact of the transanal approach on long-term functional outcomes requires further research [84].

While the Ta-IPAA is considered safe and feasible, and early experiences suggest comparable short and medium-term functional results to traditional approaches, there is a need for additional comparative outcomes data and a better understanding of the ideal training and implementation pathways for this procedure [83,85,86,88,89,90].

## 7. Complications and Functional Outcomes

Studies have indicated that IPAA patients are at increased risk of short-term pouchitis, wound infection, bowel obstruction, ileus, sepsis, anastomotic leak, and fistulas to the pouch. The most frequent long-term complications are fecal incontinence, small bowel obstruction, sexual dysfunctions, and pouch-related complications (fistula, chronic pouchitis, cuffitis, Crohn’s disease, small-volume pouch, and irritable pouch) [24]. Among these, pouchitis is the most common complication that seriously impairs long-term prognosis, even after 30 years from the pouch construction, leading to substantial morbidity and occasionally pouch failure [92].

Risk factors for developing pouchitis include a short J pouch, recurrent ulcerative colitis, and a preoperative high white blood cell count [93]. The length of the J pouch is also associated with the development of late post-IPAA complications. A more extended pouch may be a better surgical option for preventing complications such as increased defecation frequency and pouchitis. 

The postoperative mortality rate is 0.1% [67]. Despite the rates of complications, most patients achieved a good quality of life with an IPAA compared to their perioperative state [92,94].

However, certain factors, such as high stool frequency, fecal incontinence, and pouchitis, have been associated with impaired QoL [95,96,97,98,99].

The American Society of Colon and Rectal Surgeons states that sexual and urinary functions are not significantly affected postoperatively. However, some women may experience increased urinary urgency, frequency, and incontinence over time [60].

A multicenter cross-sectional study found that patients with IPAA may have higher disability scores compared to those with medically managed UC, with female sex, and public insurance being predictive of significant disability in patients with IPAA [100].

Additionally, IPAA patients have high costs related to close endoscopic surveillance of the pouch [69,70].

When considering female patients of childbearing age, we should keep in mind that the creation of a pouch is associated with an increased risk for infertility. Hence, a subtotal colectomy with delayed pouch formation may be a valid alternative until they can have children [101].

Overall, while QoL after IPAA is generally excellent and comparable to the healthy population in many domains, attention to managing complications and functional outcomes is essential for optimizing long-term QoL for these patients. A shared decision-making approach should be used to tailor procedure selection to the patient’s preference. The techniques of dissection, vascular ligation, and dissection of the mesocolon, as well as the treatment of the omentum and management of the rectal stump, are not yet defined in the clinical guidelines. The surgeon’s expertise is integral to the decision-making process by providing relevant information on the surgical options while ensuring patient preferences and the best QoL.

## 8. Conclusions

In an era of an increasing number of biological therapies and newly approved treatments, ASUC remains a life-threatening condition with elevated comorbidity. The management of ASUC remains challenging and requires close patient monitoring. Prompt recognition of symptoms and whether surgery is needed is pivotal. Despite the superiority of the minimally invasive approach over open surgery, robotic ileal pouch–anal anastomosis (IPAA) has shown comparable short-term outcomes to laparoscopy. The transanal ileal pouch–anal anastomosis has recently been applied, showing promising short- and medium-term functional outcomes. However, the impact of the transanal approach on long-term results requires more extensive studies, and outcomes need to be compared with conventional techniques. 

## Data Availability

Not applicable.
